# COVID-19 vaccine: vaccinate the young to protect the old?

**DOI:** 10.1093/jlb/lsaa050

**Published:** 2020-06-26

**Authors:** Alberto Giubilini, Julian Savulescu, Dominic Wilkinson

**Affiliations:** 1 Oxford Uehiro Centre for Practical Ethics and Wellcome Centre for Ethics and Humanities, University of Oxford, Oxford, UK; 2 Melbourne Law School, University of Melbourne, Victoria, Australia

**Keywords:** COVID-19, vaccination, prioritization, resource allocation

## Abstract

When we have a vaccine against COVID-19, who should be vaccinated first? The question is relevant because, initially, vaccine availability will likely be limited. After healthcare and some other essential workers, it might seem the most obvious candidates are the elderly and other groups that are more vulnerable to the virus. However, we argue that this is not necessarily the case. Protecting the most vulnerable might require prioritizing vaccinating children in order to maximize the benefits of indirect immunity for the elderly and the other vulnerable groups. Whether this will be the best strategy from a public health perspective will depend on characteristics of the vaccine and of the virus, which are currently unknown. Here, we assess this possibility from an ethical point of view, by drawing comparisons and analogies with the case of the flu vaccination and with other examples of health policies and practices. We conclude that there are strong ethical reasons to vaccinate the young to protect the old, provided that the risks imposed on children are reasonable, even if that implies using children as a means to protect the elderly and the vulnerable.

## A POSSIBLE VACCINE FUTURE

It is April 2021. A vaccine against COVID-19 has been tested and is now available. The last 12 months have been incredibly disrupted. Economies are in tatters, people have lost jobs, many have died, and many of those who have survived are struggling with the financial and mental health consequences of the restraining measures. Repeated cycles of relaxing and tightening social distancing have created uncertainty, to the detriment of both economic growth and mental well-being.

Even if at some point strict lockdown measures have been relaxed for certain people for the sake of economic recovery, many elderly people have been effectively trapped in their homes for more than a year. They have had friends die from the virus and have feared that they too would become unwell. They have been waiting desperately for months for the long-awaited vaccine in order to be released from ‘prison.’

Now the vaccine is here. The government, understandably, decides to make the first batch of the vaccine available to healthcare workers. When the second batch follows quickly, many expect that it would be given to the elderly. But instead, the government announces that children under 10 years would be immunized first, as part of a strategy to maximize the long-term benefits of the vaccine, given limited availability.

The decision leads to a public outcry. How could this be ethical, after all the elderly have been through and considering the risk that COVID-19 poses on them? This is the question we want to address in this paper.

## THE PROBLEM

The world is anxiously waiting for a vaccine against COVID-19. That is widely considered as the real end point of this global health emergency, when full normality can not only be restored but actually maintained. However, this is a rather optimistic view. The end point of this emergency will not be when we have a vaccine, but when we have an adequate vaccination policy to get the vaccine out to people in the most effective way.^1^ Formulating and implementing such a policy might be more difficult than it might initially seem. Which policy will best achieve the goal depends in large part on the characteristics and availability of the vaccine.

The vaccine will allow a return to full and lasting normality only if there will be high enough uptake and if it will be distributed in the most effective way. The first condition seems obvious and raises the question of whether the vaccine should be compulsory, which we will not address here. But the second risks being overlooked, for two reasons. One is that there seems to be a tendency to think that once we develop a vaccine, this will be available for everyone straightaway, which is very unlikely. Availability is likely to be limited initially. The other reason is that the general assumption is that we should give priority access to those who need the vaccine most, that is, more vulnerable groups: the elderly and those with certain preexisting health conditions. However, this might not be the most effective strategy to protect the vulnerable. A lot will depend on the characteristics of the vaccine and more specifically for which groups it will be more effective and what the risks of side effects willbe.

In this article, we want to explore the possibility that there might be public health reasons and ethical reasons to target children and young people as a matter of priority, in order to build up immunity to protect the elderly. Depending on the characteristics of the vaccine, this might mean imposing some cost on children—eg in terms of taking on the small risks of the vaccine—for the sake of vulnerable groups.

A possible vaccination policy that targets children in order to protect the most vulnerable, and particularly the elderly, might not be necessary. If we end up having a vaccine that works well on the elderly and the other vulnerable groups, then these groups should probably have priority access. Indeed, if at the point of implementation there is significantly greater confidence in safety and effectiveness in adults than there is in children, for example, if there has been significantly less experience in children testing, that will be a reason to prioritize adults over children. But what if the vaccine will not be like that? What if we have sufficient confidence in vaccine safety in children and the vaccine will be more effective at building collective immunity if administered to children rather than to adults (given initial scarce availability)?

In the second section, we are going to briefly present some of the relevant ethical issues about distribution of scarce healthcare resources in this COVID-19 pandemic. In Section 3, we will spell out how these ethical considerations apply to the case of vaccination. We will explain why, if the vaccine turns out to be more effective on children than on the elderly, we might need to target children with vaccination policies in order to create the best protection possible for the elderly, at least while vaccine availability is limited. Finally, in Section 4 we will examine some of the ethical implications of ‘using’ children as targets of vaccination policies to protect the elderly, and we will argue that the strategy could be ethically justified. We will end by spelling out how the general ethical principle about the permissibility of targeting one group as a means to achieving a broader societal benefit can have different implications for vaccination policies. These implications would also depend on whether the vaccine will turn out to have different characteristics than the ones we focus on and if we decide to prioritize different kinds of collective goods.

## ON PRIORITIZATION CRITERIA IN HEALTHCARE

It seems relatively uncontroversial that healthcare workers and other categories providing essential services should have priority access to the vaccine. First, we need their services but their work exposes them to risk of infection. Since they have a right to work in the safest conditions possible, and since, arguably, the risks associated with caring for sick patient during epidemics go beyond their normal professional responsibilities,^2^ it seems plausible that we have a duty to give them priority access. Second, they risk infecting others if they are contagious, so there are important public health considerations in favor of prioritizingthem.

But assuming essential workers ought to prioritized, who should be next? Even if we agreed that the aim is protect the most vulnerable, the means of achieving that aim is not as obvious as it mightseem.

The measure for the effectiveness of the COVID-19 vaccine and of related vaccination policies will be the number of deaths that it will be able to prevent. The high public health and economic toll of COVID-19 is caused in large part by the relatively high number of people who are dying of it and the massive amount of public health resources (eg beds in intensive care units ((ICUs), healthcare staff, etc.) required to limit the number of deaths. Reducing the number of the most life-threatening cases should be the aim of vaccination policies. Therefore, those who are more likely to suffer severe symptoms and to die are the ones whose protection should be considered a priority in order to maximize the effectiveness of the vaccine.

There are two plausible criteria for allocation of scarce health resources. One is the maximization of the overall benefit for the population—in philosophical jargon: the maximization of expected utility. The other is the prioritization of the needs of the worst off, in philosophical jargon: the prioritarian principle of distributive justice (eg^3^^,^^4^). Although these two criteria are conceptually distinct, they would often yield the same practical conclusions about resource allocation, given that usually the person who needs the resource the most will benefit the most (this consideration is indeed at the basis of prioritarianism).

However, this is not always the case. The probability of actually benefitting from the resource, and its opportunity cost, need to be taken into account. For example, in the case of allocation of beds in ICUs during the COVID-19 crisis, the criterion that some medical associations (eg in Italy) have adopted in triage decisions is prioritization not according to need, but according to chances of survival. Maximization of expected utility conflicted with the prioritarian principle. It was decided that maximization of expected utility should take priority. Thus, instead of giving the ICU bed to an 85-year-old person, who needs it more because they are more likely to die of complications of COVID-19, the decision was to give it to an otherwise healthy 40-year-old, who may need it less because they are more likely to survive without it. In this way, it is more likely that the scarce resource utilized will be effective. Or it might be argued that they should be allocated according to expected QALYs (acronym for ‘quality-adjusted life years’). According to this criterion, we should give it to an otherwise healthy 70-year-old with decent chances of survival rather than to a 50-year-old with equal or maybe higher chances of survival but with some severe disability that makes their quality of their life significantly lower, according to some plausible measure of quality of life^5^. We are not going to discuss these criteria here. However, we mention them just to point out that scarce health resources are not and should not necessarily be allocated purely according to need in order to maximize their expected benefits.

## SAVING THE GREATEST NUMBER THROUGH VACCINATION POLICIES

In the case of vaccination, if we wished to maximize the number of expected QALYs saved by a certain policy (a standard measure of effectiveness of health systems), we would need to consider probability of survival, length and quality of life. This may require prioritizing a certain group, such as those in late middle age. If, instead, we assume the goal is simply to save the greatest number of lives (regardless of length or quality), which is the rationale behind the current lockdown measures, it would seem that the goal would be best achieved by prioritizing the elderly, say those over the age of 60.

However, even if the aim is simply to save the greatest number, prioritizing the elderly might not be the best strategy.

How to best protect those who most need protection and save the greatest number will depend on the characteristics of the vaccine. One of the benefits of vaccination is that it often protects not only those who receive it but those around them as well. We often have an extra ‘weapon’ when it comes to distributing vaccines, which we do not have when distributing other kinds of scarce healthcare resources. Vaccination strategies can aim at creating as much immunity as possible by relying ‘both’ on the individual and the collective benefit of the vaccine. An ICU bed might prevent one death, but one dose of a vaccine can prevent many. So in the case of vaccination, it is important to figure out how to effectively create immunity at the collective level.

On the basis of this, there might be a lesson to be learned from the flu vaccine. Seasonal flu kills up to 650,000 people worldwide every year, the vast majority of whom are over 65^6^. In being significantly more dangerous for the elderly, the flu is similar to COVID-19. It has traditionally been considered important that the elderly and others particularly vulnerable to infection (eg those with chronic respiratory diseases) be vaccinated against the flu (eg^7^). However, targeting the elderly in vaccination policies has not maximally reduced influenza-related mortality rates. An increased uptake of the flu vaccine among the elderly has not consistently correlated with a decline of flu-related deaths in the same population.^8^ Relying on direct protection through vaccination is not necessarily the most effective way of protecting the vulnerable.

Flu vaccine efficacy decreases with age, at least after the age of 60, and on people over 70, the efficacy might be as low as 23 percent.^9^ The elderly are generally less immunocompetent. Although children are less likely to experience severe consequences and to die of flu, they tend to get infected more frequently, to remain infected for longer^10^, and therefore to become vectors for the contagion of the elderly, who are not adequately protected by the vaccine.

This means that vaccinating children is a more effective strategy to build up immunity at the collective level than vaccinating the elderly, at least with respect to flu. Children are not the ones who need the vaccine the most, because they are less likely to suffer severe consequences from the flu than the elderly. However, they are the ones who can mostly contribute to the effectiveness of the vaccine at the population level and to protect those who need protection the most. We have argued elsewhere that flu vaccination strategies should target children in order to protect the elderly.^8^

Thus, allocating vaccines in a way that benefits the most vulnerable and that maximizes the vaccine’s effectiveness might mean prioritizing or targeting those who are less likely to benefit from the vaccines or that need the vaccine the least. Whether this will be the case with the COVID-19 vaccine will depend on features of the vaccine which we do not know yet. More specifically, it will depend on whether the vaccine will be more or less effective on young people and on whether the side effects will be more or less likely or severe in young people. If the vaccine turns out to be as effective or more effective on the elderly, the presumption is that they should be given priority access, other things being equal, given that they are more at risk. If the vaccine turns out to be more effective on children, then saving the greatest number might require prioritizing children, other things being equal (eg given the same level of risks in both population and perhaps even a slightly higher level of risk on children).

It will also depend on how contagious COVID-19 actually is for young people. This last consideration is important to determine to what extent children can be a threat for the elderly and the vulnerable. It is uncertain how contagious this coronavirus is in a pediatric population. The widespread perception at present is that COVID-19 affects children much less than other population groups. This is because relatively few children have died of COVID-19-related complications or have developed severe symptoms or indeed have developed any symptoms at all. According to a study recently published in the *Journal of the American Medical Association*, for instance, less than 1 percent of recorded COVID-19 cases in China were in children younger than 10.^11^ However, one credible hypothesis is simply that children develop symptoms less frequently because their body reacts better to the virus once they get infected, rather than because they get infected less frequently.^12^^,^^13^ More research needs to be done on this, but if that were the case, it would mean that children are very subtle vectors of the disease. As they cannot be easily identified as sources of contagion and isolated, they represent a big threat for the most vulnerable. In the case of the flu vaccine, one reason for targeting children is that children are more likely to infect others because they are more likely to get infected. In the case of COVID-19, it might well be that children are more likely to infect others in large part because their infection is less likely to be detected. If that were the case—and this is something at the moment we are uncertain about—having a vaccination policy that targets children would likely be the most effective.

Thus, depending on a number of variables, indirect immunity might well confer more protection to the elderly and the other vulnerable population groups than direct vaccination. If that were the case, would it be ethical to implement a vaccination policy that targets children in order to protect the elderly?

## THE ETHICS OF TARGETING THE YOUNG TO PROTECT THE OLD

What kinds of ethical concerns might be raised by a policy that prioritizes children access to the COVID-19 vaccine in order to protect the elderly? Before we address them, consider the following two cases.

### Folic acid intake in pregnant women

It is widely accepted that it is at least permissible, if not morally required, for women to get an adequate intake of folic acid when pregnant. Folic acid can prevent serious risk of disease and disability in their future children, such as spina bifida. Folic acid is not only recommended but typically added to basic everyday food, such as breakfast cereal and bread. This is normally seen as unproblematic. The actual benefit of folic acid for women, and for people in general, is minimal unless there are specific health conditions, such as folate deficiency anemia. Actually, there are cases in which folic acid is contraindicated. For example, in rare cases people can experience allergic reactions including anaphylaxis.^14^ And yet, we commonly accept that it is at least permissible to encourage the introduction of a certain substance into a person’s body in order to benefit someoneelse.

### Pediatric bone marrow donation

Donating stem cells to a sibling or a relative through bone marrow donation is a commonly accepted practice, including when the donor is a child. This kind of donation is often necessary to save the recipient’s life, for example, in cases of leukemia where other compatible donors cannot be found in a timely manner. The practice is invasive and requires general anesthesia. And although bone marrow harvesting is considered relatively safe, there are some risks of nerve, bone, and tissue damage, as well as the risks normally associated with anesthesia (including death). The procedure is considered ethically acceptable even if donor children are not legally competent to make these kinds of decisions.^15^

The case of vaccinating children to protect the elderly is similar to these two cases in some relevant respect. In all of these cases we are targeting a certain individual in order to benefit someone else. In all of these cases, the main benefit accrues to the third party and not to the individual who is ‘used’ to benefit others. Although there might sometimes be some benefits to the direct ‘target’ of the intervention (such as to the mother in the folic acid case), there are also risks associated with it. The risk assessment is clearly not favorable to the direct target in the case of the bone marrow donation. And although the target’s legally valid consent to the procedure is not always possible (such as in the bone marrow donation case), the intervention is generally considered permissible as long as it is done in a way that appropriately balances the target’s interests, that is, that the risks are reasonable. Reasonable risk in this context means that the benefit could not be accrued in another less risky way and that the risks are minimized and proportionate to the combined benefits to the child and others. The absolute risk should be such that the sacrifice constitutes an “easy rescue”, that is no more than could otherwise be imposed on a child in ordinary life.^16^

From all these examples, two main ethical issues arise: treating someone as a (mere) means in order to benefit someone else and targeting one person to benefit someone else without the target’s autonomous consent.

### Using children as means?

One concern with vaccination policies targeting children to benefit (primarily) the elderly and the most vulnerable might be that we are ‘using’ children as means to protecting the elderly. More generally, we are using children to benefitting the collective through alleviating the burden on healthcare systems. The greatest benefit of vaccinating them *en masse* will accrue to the vulnerable ones through indirect protection, rather than to the vaccinated children themselves.

The main problem with this strategy is that no vaccine is 100 percent safe and 100 percent effective. The COVID-19 vaccine will be no exception. Human trials will need to demonstrate an acceptable level of safety and effectiveness on various population groups. The vaccine currently being developed at the University of Oxford, for instance, is now entering phase II and III trials, and phase II includes testing on children aged 5–12.^17^ Thus, safety and effectiveness will be tested in this age group. As with all vaccines, however, trials will never guarantee 100 percent safety and 100 percent effectiveness. The ethical issues that we are discussing in this paper would not be as pressing if we could have a vaccine that was 100 percent and 100 percent effective. The reason why the problem can be framed in terms of treating children as ‘means’ is that, under this strategy, in fact we would be exposing them to some level of risk, however small it is, also or even primarily for the sake of protecting others. The children will vastly benefit from the vaccine because they will gain immunity, but as with all vaccines, the risk is notzero.

The situation may be worse with COVID-19 as the problem is so urgent and no vaccine has ever been developed against a coronavirus (of which COVID-19 is a type), although past research on SARS and MERS vaccines has identified potential approaches (Mayo Clinic 2020).

The challenge requires that a new type of vaccine be developed. Research has been made faster by new technologies being deployed, such as a new adenovirus manufacturing platform adopted by Oxford University, as well as next-generation gene sequencing methods.^18^ But the need to adopt and use somehow novel methods and to have the vaccine as quickly as possible means that the vaccine may be entering unchartered waters. There is a chance that, although still very safe, the risks of this vaccine be greater than in the case of more conventional vaccines.

Moreover, the benefit to children may be significantly less. Mortality rate in children has been consistently around 0.01 percent, which is similar to the flu’s mortality rate in children.^19^ If the vaccine provides benefits to children, they are likely to be marginal, especially compared to the benefits provided to the elderly. Thus the issue of whether vaccinating children is using them as means arises.

It might be thought that using individuals in this way is morally wrong. However, using people as means is not morally problematic *per se*. We do that in many everyday interactions when we require the service of other people. We do that when we encourage pregnant women to take folic acid or harvest bone marrow from certain children to save the lives of their siblings. Using people as means can be morally wrong only if people are used as means in a way that harms them or that is in some other way disrespectful of them. Even in that case, it could be plausibly argued that treating them as means is morally wrong only if they do not consent to it. This might be rephrased, consistently with one formulation of the Kantian categorical imperative, by saying that it is morally impermissible to treat people as ‘mere’ means. As Kant put it, ‘[s]o act that you treat humanity, whether in your own person or in the person of any other, always at the same time as an end, never merely as a means.’^20^

Now, there are some extreme circumstances in which even treating people as means in a way that is harmful or disrespectful to them and without their consent may be considered permissible. The prohibition against treating people merely as means weakens where something sufficiently valuable is at stake. Those familiar with the thought experiments related to the ‘trolley problem’ in moral philosophy would easily recall some variations of the original scenario in which it seems intuitively permissible to kill one individual in order to save five. Take ‘loop case’ variation of the original trolley case scenario, for instance. Diverting a trolley onto a sidetrack where it would kill one person, instead of onto a sidetrack where it would kill five, seems intuitively permissible even if the two tracks are connected by a loop, whereby, if the one person was not there to stop the trolley, the trolley would go around the loop and come back to kill the five. In this case, the one person is in fact used as a means to save the five. This seems intuitively permissible to many even if it means that the one person dies. So it is not so obvious that even using people as *mere* means is always morally wrong. It might be plausibly argued that this is what we do when we harvest bone marrow from children to save their siblings.

In the case of the COVID-19 vaccine, what is at stake is very valuable—that is, the life of many vulnerable people. So perhaps it would not even be wrong to treat children as mere means. Unlike the fictitious loop case we have just described and unlike the bone marrow case, the sacrifice we are asking young people is vastly smaller. All they would need to do is get vaccinated and accept the very small risks of vaccination, which are vastly smaller than in the fictitious loop case and likely smaller than, or at worst similar to, the risks in the case of bone marrow donation. In the case of vaccinating children for the flu, the vaccine actually benefits children as a small proportion do suffer significant consequences and even die.^6^ The vaccine is comparatively safe. If the COVID-19 vaccine is similar to flu vaccine, then by ‘using’ children as a means to protect the elderly and the vulnerable, we are using children as means in a way that also actually benefits the children, as they acquire protection against COVID-19. Even if COVID-19 seems to be not life-threatening for young children, it still is a disease that can cause some complications and some level of pain or discomfort. And however small, there is some risk of severe complications and death even in children.

Thus, whether it is reasonable to use children will be determined by the vaccine’s safety in children and their rate of complications from both COVID-19 and vaccination. If the profile were similar to flu, it would be reasonable to vaccinate children primarily to benefit the elderly because the risk is reasonable.

If we think bone marrow donation is permissible even if we are treating one child as a means to saving another one, and that encouraging women to take folic acid is permissible even if we are doing it just for the sake of the future child, then we should think that vaccinating children to protect the elderly is permissible, as far as our concern is about using someone as a means. It is true that in the bone marrow case and in the folic acid case, one might think that there are special obligations toward family members that require certain sacrifices of people. However, when the sacrifice required of individuals is very small, the case for requiring these sacrifices is very strong even in the absence of special obligations. When the sacrifice actually entails some benefits, the case for requiring it is even stronger.

### Autonomy

A second ethical concern is that children cannot autonomously choose vaccination. There can be two versions of this objection, depending on how coercive the vaccination policy will be. If we adopt some coercive COVID-19 vaccination policy, then there is an autonomy-based objection that applies to coercive vaccination policies more generally. This would require a separate discussion about the relative weight of autonomy (either bodily autonomy or parental autonomy) compared to considerations about public health benefits of vaccination and fairness in contributing to the public good of herd immunity. We have argued elsewhere that such considerations weigh in favor of compulsory vaccination (eg^21^^,^^22^^,^^23^). However, in this paper we want to focus more specifically on targeting children in the future COVID-19 vaccination policy, regardless of whether the policy will be coercive. If there is any concern about autonomy specifically with regard to children as a target of vaccination policies, then it would be about children’s autonomy. It is exactly the same autonomy-based concern that arises in the child bone marrow donationcase.

However, this concern can be dismissed by pointing out that children are not generally deemed competent to make autonomous decisions about health care or disease prevention anyway. We normally accept that parents make such choices on their behalf. Targeting children cannot meaningfully infringe on their autonomy simply because children do not have the relevant capacity for autonomous decisions—that is, whatever capacity for autonomous decision they have, it is normally not taken to warrant ‘a right to’ autonomous decision. Hence, targeting children in vaccination policies cannot infringe upon their autonomy in an ethically relevant way. Simply, there is no autonomy to be infringed upon. Of course, older adolescents who are competent would be an exception, but in the case the relevant autonomy-based objection would be the one often raised against compulsory vaccination. As we said, we are not going to address ithere.

If we think that child bone marrow donation is permissible even if children cannot provide valid, that is autonomous, consent, then we should think that targeting children in vaccination policies is permissible, too, as far as our concern is about autonomy.

Thus, if it will turn out that a vaccination policy targeting children is more effective at creating immunity at the collective level, and if initial vaccine availability will be limited, there seem to be significant reasons for prioritizing child access to the vaccine. This measure would be more likely to be effective, and there would be no ethical objection to this kind of policy, as far as we cansee.

In a way, this is what is already happening. Children and young people, and society more broadly, are paying a high cost for the sake of protecting the vulnerable. Children are forced to keep social distance from their peers, they cannot attend school, they are prohibited from accessing playgrounds, and they will inevitably pay a high price from the huge economic impact of the lockdown at present and in the future. While the elderly and vulnerable people are the main victims of the virus, young people, and children particularly, are arguably among the main victims of the social measures implemented. Their life is going to be significantly impacted, in a negative way, by these measures. And they have less to gain from such measures, given that they are very unlikely to experience significant symptoms and complications and extremely unlikely to die of COVID-19. The limitations they are subject to are meant to benefit the elderly and the most vulnerable groups primarily. This is the background against which the post-vaccine scenario and the ethical issues around the distribution of the vaccine must be assessed. It may be that the sacrifices of being vaccinated for a child are smaller than the sacrifices of lockdown.

## CONCLUSION

The proposal to vaccinate they young to benefit the old is controversial. We need to start debating it now, in the cool, calm hour, as Butler putit.

There is a scientific as well as an ethical challenge to be addressed before adequate law can be formulated. Ethical discussion needs to commence, and legal options and possible plans need to be examined now. In this way, when we have a vaccine, we will be able to get it out to the right people and to return to full and lasting normality as soon as possible.

When the UK government started to seriously consider various options to control the spread of COVID-19 in early March 2020, many people at some point had the impression that the strategy was that of building up natural herd immunity. This strategy would have gone in the exact opposite direction of that pursued by most other countries, which enforced harsh lockdown measures at an early stage, with the exception of Sweden. Some statements by some representatives and scientific advisors of the government seemed to suggest that this was the line of the government, at least initially, because it was considered the most effective way to keep the number of infections at any one time within the capacity of the NHS to ‘absorb’ new cases.^24^ Whether that strategy would have been effective, or more effective than the lockdown enforced a few days after, is unknown. According to some predictions, it would have led to an estimated half million deaths before the vaccine becomes available, way more than the ones we are likely to have with a lockdown. Many thought that this strategy would have been unethical because it would have meant exposing certain people to considerable risks of illness and deaths for the sake of protecting others. According to their view, you cannot make certain people sick in order to protect others through immunity at the collective level.

We do not take a stance on the ethics of that kind of policy here. Rather, we want to point out that the strategy of vaccinating children in order to protect the vulnerable would follow the same logic that seemed to inform the initial attitude of the UK government: building up immunity at the collective level in order to protect the vulnerable. However, it would allow that objective to be achieved without anywhere near the risks and the ethical costs that many identified in that natural herd immunity strategy. If building up immunity in a way that would protect the vulnerable is desirable, an appropriate use of the vaccine would allow to achieve that aim at no significant ethicalcost.

There remain important empirical questions to be answered, both in terms of the role of children in spreading COVID-19 and the effectiveness of any candidate vaccines in different groups. However, if the COVID-19 vaccine does turn out to be significantly more effective on children, and children represent a key vector for spread, then there is a strong ethical case that vaccination policies ought to prioritize children rather than the elderly.

Importantly, the same ethical point we have made in this paper can have different practical implications depending on the other groups that might be or become higher risk groups, the kind of good we want to achieve, the best way to achieve it, and the characteristics of the vaccine. Here we have only discussed what seems one of the most feasible and effective instantiations of a more general principle that could guide vaccination decisions and policies in the next months. But there are other possible applications of the same principle. For instance, prisoners are a higher-risk groups because of the higher risk of infection in jails’ closed environments, and retail shop workers will become a higher-risk group once the lockdown measures are eased. Some of these groups are also essential for restarting the economy and therefore for bringing about important social goods, beyond the mere contribution to realizing collective immunity. These are all considerations that need to be taken into account when deciding which groups to target in order to protect which other groups and to contribute to the collective good more generally. Importantly, the decision about how to balance these different considerations will depend on the vaccine’s features. For example, there is a possibility that one of the most promising vaccine candidates will be effective at preventing people from getting sick, eg from developing pneumonia, but not too effective at stopping transmission, because the virus might still be present in the nose, as shown in animal experiments and spread through sneezing or coughing.^25^

Once we take all these variables into account, we might still want to prioritize those who do not stand to benefit the most from the vaccine, if there is some important societal good to be achieved by doing this. But we will need to decide whom to prioritize, for the sake of whom, and in view of achieving which societal good. For example, we might want to prioritize those groups who are essential to the upkeep of society, such as factory workers, grocery store workers, medical and nursing school students, and others. It all depends on what kind of good we want to achieve (eg protecting the vulnerable, restarting the economy, longer-term economic benefits, etc.) and what is required to achieve it (eg creating herd immunity, allowing the workforce to safely and quickly return to full activity, etc.). The relative ethical weight of the different goods at stake and their opportunity costs would need to be considered for each of these possible solutions. Our point is that, assuming the vaccine will be safe and effective, prioritizing a certain population group (also) as a means for achieving a larger societal benefit is ethically permissible and might indeed be ethically required.

## FUNDING

A.G.'s work was funded by UKRI/AHRC grant AH/V006819/1 and by the Wellcome Trust, grant numbers WT104848 and WT203132. J.S.'s work was funded by the Wellcome Trust, grant numbers WT104848 and WT203132. D.W.'s work was funded by the Wellcome Trust, grant number WT203132.

## Footnotes

1 It is worth noting that even when the vaccine comes, if COVID is anything like influenza, it’s likely to continue to mutate, and vaccines will be only partly effective. The emergency may be over, but the damage, especially to immunocompromised people, is likely to continue for years.

2 Malm, H. Thomas May, Leslie P. Francis, Saad B. Omer, Daniel A. Salmon & Robert Hood, Ethics, Pandemics, and the Duty to Treat, *Am J Bioeth*, 8 8, 4–19 (2008).

3 Parfit, D. (2000) Equality or priority. In: Clayton, M., Williams, A. (eds.), *The Ideal of Equality. Delivered as the Lindley Lecture at the University of Kansas in 1991*. Houndmills: Palgrave, pp. 81–125.

4 Parfit, D. (2012) Another defence of the priority view. *Utilitas*, 24: 399–440.

5 Savulescu J., Cameron J. & Wilkinson D. Equality or utility? Ethics and law of rationing ventilators, *Br J Anaesth*, 125 (1): 10–15 (2020). https://doi.org/10.1016/j.bja.2020.04.011.

6 WHO. (2020). *Seasonal Influenza*, http://www.euro.who.int/en/health-topics/communicable-diseases/influenza/seasonal-influenza, accessed Apr. 17, 2020.

7 OECD. (2016). *Influenza Vaccination Rates [Online]. OECD Data*, https://data.oecd.org/healthcare/influenza-vaccination-rates.htm.

8 Bambery, B., Thomas D., Michael J. Selgelid, Hannah M., Alberto G., Andrew J. Pollard, Julian S., Influenza Vaccination Strategies Should Target Children, *Public Health Ethics*, 11 (2), 221–234 (2018).

9 Govaert, T. M., Thijs, C. T. M. C. N., Masurel, N., Sprenger, M. J. W., Dinant, G. J. and Knottnerus, J. A. The efficacy of influenza vaccination in elderly individuals: a randomized double-blind placebo-controlled trial. *JAMA*, 272 (21), pp. 1661–1665 (1994).

10 Glezen, W. P. (2006) Herd Protection Against Influenza. *Journal of Clinical Virology*, 37: 237–243.

11 Wu Z. & McGoogan J.M. Characteristics of and important lessons from the coronavirus disease 2019 (COVID-19) outbreak in China: summary of a report of 72 314 cases from the Chinese Center for Disease Control and Prevention. *JAMA*, 323 (13): 1239–1242 (2020 February 24) (Epub ahead of print).

12 CDC, Coronavirus Disease 2019 in Children—United States, February 12–April 2, 2020. *MMWR Morb Mortal Wkly Rep*, 69, 422–426 (2020). doi:10.15585/mmwr.mm6914e4.

13 Lu et al. SARS-CoV-2 Infection in Children, *N Engl J Med*, 382: 1663–1665 (18 March 2020).

14 NHS. (2019). *Folic Acid*, https://www.nhs.uk/medicines/folic-acid/, last accessed 6 July 2020.

15 Bendorf, A. & Kerridge, I.H.. Ethical issues in bone marrow transplantation in children. *J Paediatr Child Health*, 47 (9), 614–619 (2011).

16 Savulescu, J. & Hope, T. 2010. Ethics of Research. *In The Routledge Companion to Ethics* (J. Skorupski ed). Abingdon: Routledge, pp. 781–795.

17 See updates at http://www.ox.ac.uk/news/2020-05-22-oxford-covid-19-vaccine-begin-phase-iiiii-human-trials (accessed May 25, 2020).

18 For an account, see Rafiq and Micheletti’s article in the Conversation, https://theconversation.com/coronavirus-vaccine-search-how-were-preparing-to-make-enough-for-the-whole-world-137970 (accessed May 28, 2020).

19 RCPCH Research & Evidence Team. *Covid-19-Research Evidence Summaries*. Royal College of Paediatrics and Child Health. (2020).

20 Kant, I. (1785). *Grundlegung zur Metaphysik der Sitten. Translated as Groundwork of the Metaphysics of Morals in Immanuel Kant: Practical Philosophy*. (Mary Gregor ed.) Cambridge: Cambridge University Press, 37–108.

21 Giubilini, A. An Argument for Compulsory Vaccination: The Taxation Analogy. *J Appl Philos*, doi:10.1111/japp.12400 (2019a).

22 Giubilini, A. *The Ethics of Vaccination*, Palgrave MacMillan (2019b).

23 Giubilini, A. Julian Savulescu, Vaccination, Risks, and Freedom: The Seat Belt Analogy, *Public Health Ethics*, 12, 3, 237–249 (2019).

24 See eg the *Guardian* Report, https://www.theguardian.com/world/2020/mar/13/herd-immunity-will-the-uks-coronavirus-strategy-work).

25 Doramalen, van Neeltje et al. 2020. *ChAdOx1 nCoV-19 vaccination prevents SARS-CoV-2 pneumonia in rhesus macaques, pre-print not peer-review*. https://www.biorxiv.org/content/10.1101/2020.05.13.093195v1.full.pdf (accessed May 26, 2020).

